# The Neutrophil–NET Axis in Immune Checkpoint Inhibitor Resistance in Non-Small Cell Lung Cancer: Roles, Biomarkers and Therapeutic Opportunities

**DOI:** 10.3390/biom16030400

**Published:** 2026-03-08

**Authors:** Geng Xu, Bing Wang, Elisa Giovannetti

**Affiliations:** 1 Department of Medical Oncology, Cancer Center Amsterdam, Amsterdam UMC, Vrije Universiteit, 1081 HV Amsterdam, The Netherlands; g.xu1@amsterdamumc.nl (G.X.); b.wang1@amsterdamumc.nl (B.W.); 2 Cancer Pharmacology Laboratory, Fondazione Pisana per la Scienza, Via Ferruccio Giovannini 13, 56017 Pisa, Italy

**Keywords:** non-small cell lung cancer, immune checkpoint inhibitors, neutrophils, neutrophil extracellular traps, resistance, tumor microenvironment

## Abstract

Non-small cell lung cancer (NSCLC) remains a leading cause of cancer-related death. Although molecular stratification and multimodal therapy have improved outcomes in selected patients, overall prognosis is still limited by late diagnosis, heterogeneity, and treatment resistance. Immune checkpoint inhibitors (ICIs) have substantially improved survival outcomes in a subset of patients; however, the overall benefit remains limited, and both primary and acquired resistance are common. Neutrophils, as key effectors of innate immune responses, can be activated by diverse stimuli and release neutrophil extracellular traps (NETs). Growing evidence indicates that neutrophils and NETs contribute to remodeling of the tumor microenvironment (TME) in NSCLC, promoting resistance to ICIs. This review systematically summarizes the biological features, key molecular pathways, and inducing factors of neutrophils and NETs in lung cancer and synthesizes evidence supporting their roles as biomarkers of ICI efficacy and prognosis. We further focus on the mechanisms by which NETs mediate immunosuppression and foster an immune-excluded TME, thereby driving resistance to immunotherapy. In addition, we outline potential therapeutic and combination strategies targeting neutrophils and NETs, providing a theoretical basis for developing optimized immunotherapy approaches for NSCLC that target neutrophils and NETs.

## 1. Introduction

Lung cancer remains one of the leading causes of cancer-related mortality worldwide, and non-small cell lung cancer (NSCLC) accounts for approximately 80–85% of all lung cancer cases [1,2]. Owing to insidious early symptoms, inadequate screening, and pronounced disease heterogeneity, many patients are diagnosed at a locally advanced or metastatic stage, and overall prognosis remains suboptimal. In recent years, progress in molecular stratification and multimodal treatment has accelerated a transition in NSCLC care toward multidisciplinary, risk-adapted, and individualized management [2,3,4]. Current treatment strategies include surgery, radiotherapy, chemotherapy, targeted therapy, anti-angiogenic therapy, and immunotherapy. For early-stage and selected locally advanced disease, surgery with perioperative treatment remains the main curative option, whereas unresectable or advanced NSCLC relies primarily on systemic therapy [2,3,4]. Although targeted therapies for molecularly defined subsets, (e.g., for EGFR- or KRAS-altered tumors) have substantially improved outcomes, and anti-angiogenic strategies provide benefits in selected patients, overall clinical gains remain limited by treatment resistance and restricted applicability across the broader NSCLC population [2,3,4].

Immune checkpoint inhibitors (ICIs) have substantially reshaped the therapeutic landscape of NSCLC. In particular, therapies targeting the PD-1/PD-L1 axis have become integral components of treatment for advanced disease, selected locally advanced settings, and perioperative strategies, while CTLA-4-based approaches have value in specific patient groups and regimens [3,5,6]. Despite durable survival benefit in a subset of patients, overall efficacy remains highly heterogeneous, with frequent primary non-response and the development of acquired resistance after an initial benefit. This highlights an incomplete understanding of the biological basis underlying differential immunotherapy responses and underscores several major unmet clinical needs [5,6,7]. First, the mechanisms of ICI resistance remain insufficiently defined, especially the interplay between tumor cell-intrinsic alterations and immunosuppression within the tumor microenvironment [7,8]. Second, currently available clinical biomarkers show limited ability to predict durable benefit, stratify resistance risk, or inform rational combination strategies, underscoring the need for biomarkers that are more robust, reproducible, and clinically actionable. Third, the development and optimization of mechanism-guided combination regimens to increase response rates and extend the durability of benefit remains a central priority for ongoing research and clinical translation [8,9,10].

Accumulating evidence indicates that neutrophils represent a critical interface linking tumor-associated chronic inflammation, innate immune reprogramming, and resistance to immunotherapy [11]. Under persistent inflammatory cues and tumor-derived signals, activation of pattern-recognition receptors driven by pathogen-associated molecular patterns/damage-associated molecular patterns can sustain the upregulation of inflammatory mediators such as IL-1β, IL-6, TNF-α, and CXCL8, thereby promoting neutrophil recruitment, activation, and phenotypic remodeling [12,13]. In this process, neutrophils may shift from relatively antitumor states toward immunosuppressive and protumor phenotypes, contributing to tumor progression and immune escape through inflammatory amplification, extracellular matrix (ECM) remodeling, pro-angiogenic activity, and broader immunomodulatory effects [9,10]. In NSCLC, elevated neutrophil levels are frequently observed in both tumor tissues and peripheral blood, and neutrophil-related indices (e.g., the neutrophil-to-lymphocyte ratio, NLR) are closely associated with limited ICI benefit and unfavorable outcomes. Meanwhile, neutrophil extracellular traps (NETs), which are extracellular DNA protein lattices released by activated neutrophils, can facilitate tumor cell adhesion, invasion, and metastatic dissemination [11,14,15]. In addition, NETs may compromise CD8^+^ T-cell and natural killer-cell effector functions by establishing local physical barriers, concentrating immunosuppressive mediators, and thereby attenuating antitumor immune activity [11,14,15]. Clinical and translational studies further suggest that elevated circulating or intratumoral NET levels correlate with poor immunotherapy responses and worse survival, supporting the concept that neutrophil phenotypic rewiring and NET-driven inflammatory amplification may constitute an important mechanistic basis of ICI resistance in NSCLC [16,17].

Although prior studies have provided important foundations for understanding the roles of neutrophils and NETs in cancer, the existing literature has largely focused on inflammatory biology or pan-cancer perspectives [11,14,18]. Further refinement is warranted in the specific clinical context of ICI resistance in NSCLC. Moreover, there remains a need for a more integrated synthesis connecting mechanistic insights, clinical associations, biomarker translational value, and potential intervention strategies. In this review, we systematically summarize how the neutrophil–NET axis reshapes the tumor microenvironment (TME) and contributes to ICI resistance in NSCLC, highlight relevant clinical evidence and mechanistic frameworks, evaluate neutrophil- and NET-related readouts as candidate biomarkers for response prediction and resistance stratification, and discuss combination strategies targeting neutrophils/NETs and key directions for future research, with the goal of informing efforts to optimize NSCLC immunotherapy outcomes.

## 2. Biology and Heterogeneity of Neutrophils in NSCLC

Neutrophils are the most abundant leukocyte subset in peripheral blood and are primarily generated through granulopoiesis in the bone marrow [19,20,21]. Under both homeostatic and inflammatory conditions, they can be rapidly mobilized into the circulation and, guided by chemokine gradients, extravasate across the endothelium to migrate into tissues, where they execute a broad repertoire of innate effector functions, including phagocytosis, degranulation, and reactive oxygen species-associated antimicrobial and immunomodulatory activities [19,20,21]. Accumulating evidence indicates that neutrophils are markedly enriched in the TME of NSCLC [22,23]. Multiparametric flow cytometry and immunohistochemical analyses have shown that neutrophils often represent a numerically dominant immune population among tumor-infiltrating leukocytes, and independent cohorts consistently demonstrate significantly increased neutrophil accumulation in tumor regions compared with paired normal lung tissue [22,23,24]. These findings support the concept that tumor-associated chronic inflammation and sustained chemokine networks continuously drive neutrophil recruitment and retention in NSCLC [22,23,24]. Moreover, immune cell composition differs systematically across NSCLC histology. In general, lung adenocarcinoma tends to exhibit relatively greater lymphocyte infiltration, whereas lung squamous cell carcinoma is more frequently characterized by a myeloid-dominant landscape with higher proportions of neutrophils and tumor-associated macrophages. Such immunologic differences further underscore the importance of interrogating neutrophil biology when dissecting NSCLC immune ecosystems [22,23,25].

Historically, neutrophils were viewed as terminally differentiated innate immune cells with relatively uniform functions. However, studies in cancer and other chronic inflammatory contexts have established that neutrophils display substantial lineage heterogeneity and functional plasticity [11,24,26]. In peripheral blood, density gradient separation broadly distinguishes high-density neutrophils (HDNs) from low-density neutrophils (LDNs). HDNs sediment within the granulocyte fraction and largely correspond to classically defined mature neutrophils, whereas LDNs co-sediment with the peripheral blood mononuclear cell layer and are expanded in cancer, chronic inflammation, and autoimmune diseases [26,27,28]. Functionally, LDNs more often exhibit immunosuppression-associated features, including suppression of T-cell proliferation and effector activity, together with enhanced production of immunomodulatory mediators and oxidative stress-related factors, whereas HDNs are more closely aligned with canonical antimicrobial and acute inflammatory responses [26,27,28].

Within tumor tissues, neutrophils are commonly referred to as tumor-associated neutrophils (TANs). Drawing on the macrophage M1/M2 polarization paradigm, early studies proposed an analogous framework for TANs, describing an N1-like state with relatively greater antitumor activity and an N2-like state characterized by enhanced protumor functions and immunosuppressive properties [29,30]. In parallel, the concept of myeloid-derived suppressor cells (MDSCs) emerged to describe pathologically activated myeloid populations that expand in cancer and other chronic inflammatory settings; within this framework, the granulocytic or neutrophil-like subset is commonly termed polymorphonuclear MDSCs (PMN-MDSCs) and is defined by potent T-cell-suppressive activity [29,30,31]. Notably, PMN-MDSCs overlap phenotypically and functionally with immunosuppressive TAN programs and are often considered to represent the most suppressive end of the neutrophil continuum within the tumor microenvironment [29,30,31]. Importantly, the N1/N2 framework should be interpreted as a spectrum rather than a strict dichotomy. Neutrophil states can vary across spatial niches within the same tumor and shift dynamically with disease stage, inflammatory intensity, cytokine networks, and therapeutic pressure, reflecting context-dependent and potentially reversible polarization programs [24,29,30].

Neutrophil enrichment in NSCLC is not a stochastic event but is increasingly linked to molecular subtypes and co-mutation backgrounds. Growing evidence suggests that specific oncogenic programs preferentially shape a tumor immune microenvironment dominated by neutrophils and other myeloid-suppressive populations, often accompanied by limited effector T-cell infiltration or impaired function, resulting in immune-cold or immune-excluded phenotypes [32,33]. In KRAS-mutant lung adenocarcinoma, KRAS-driven signaling not only reprograms tumor intrinsic pathways but also modulates cytokine and chemokine outputs that promote recruitment of immunosuppressive populations and restrict productive T-cell infiltration, thereby establishing an overall suppressive immune ecosystem [32,33,34]. Beyond KRAS alone, recurrent co-alterations such as loss of STK11 (LKB1) and mutations in KEAP1 have been repeatedly associated with immune evasion and enhanced myeloid inflammation [32,33,34,35]. Mechanistic studies indicate that STK11 deficiency can be accompanied by increased recruitment of CD11b^+^Gr 1^+^ tumor-associated neutrophils together with reduced T-cell infiltration, while KEAP1 mutations likewise perturb immune composition and favor immunosuppressive phenotypes in lung adenocarcinoma [34,35]. Collectively, these driver and co-mutation contexts converge on a TME characterized by myeloid predominance and constrained effector T-cell activity, highlighting neutrophil-centered immune ecology as a plausible determinant of heterogeneity in NSCLC immunotherapy outcomes [32,33,34,36,37].

Emerging spatial omics and digital pathology approaches are expected to refine the characterization of neutrophil heterogeneity in NSCLC [38,39,40]. Spatial transcriptomics, for example, can resolve neutrophil-associated transcriptional programs while preserving tissue architecture, enabling the mapping of distinct neutrophil states and their spatial relationships with tumor nests, stroma, and effector lymphocytes. In parallel, multiplex immunofluorescence or immunohistochemistry combined with digital pathology can quantify neutrophil infiltration patterns and, when NET markers are included in the staining panel, delineate NET-rich immune phenotypes [38,39,40]. These spatially resolved strategies, especially when integrated with single-cell profiling, may improve patient stratification and facilitate the development of clinically actionable biomarkers [38,39,40].

In summary, NSCLC is characterized not only by increased neutrophil abundance but also by systematic alterations in neutrophil subset composition and functional states. Peripheral LDNs, immunosuppressive TAN states, and PMN MDSCs together constitute major components of a myeloid-suppressive ecosystem and exhibit dynamic plasticity under chronic inflammation and tumor-derived cues [24,29,30,31] (Table 1).

## 3. Characteristics and Functions of NETs in NSCLC

NETs are extracellular web-like structures released by activated neutrophils. They consist of a decondensed DNA scaffold decorated with histones and multiple neutrophil granule proteins, including myeloperoxidase (MPO), neutrophil elastase (NE), and cathepsin G [11,15]. NETs refers to the final extracellular “meshwork,” whereas NETosis denotes the programmed cellular process by which neutrophils undergo chromatin remodeling and release NETs in response to specific stimuli. Although NETosis was originally described as a host defense mechanism against infection, it can become persistent and pathological in cancer and chronic inflammation, representing a key manifestation of neutrophil functional reprogramming [41,42].

Based on whether plasma membrane integrity is compromised, NET release is commonly categorized into suicidal (lytic) NETosis and vital NETosis [43,44]. Suicidal NETosis is typically triggered by strong stimuli and involves chromatin decondensation, nuclear envelope breakdown, and eventual plasma membrane rupture, resulting in neutrophil death and a relatively slower kinetic profile. In contrast, vital NETosis can occur rapidly and may export DNA through vesicular mechanisms, enabling NET formation without overt membrane rupture and allowing neutrophils to retain certain effector functions [43,44]. NET DNA sources are heterogeneous: NETs most often derive from nuclear DNA but may also involve mitochondrial DNA under specific conditions. In practice, these phenotypes are better viewed as a continuum shaped by the nature and intensity of stimuli and by the local microenvironment [43,44,45].

**Figure 1:**
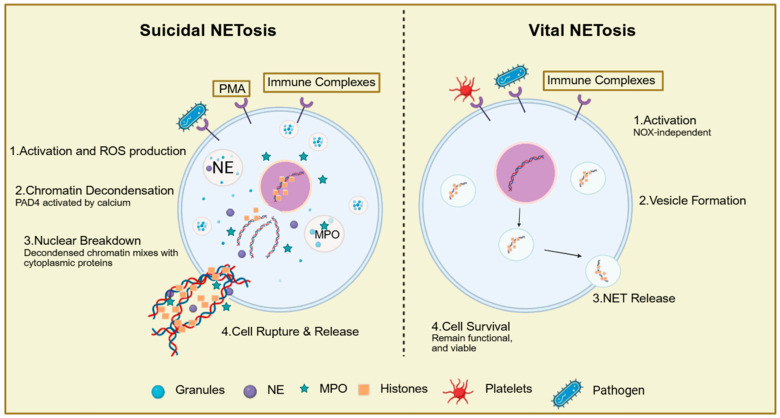
Overview of suicidal versus vital NETosis. Suicidal NETosis (**left**) is typically triggered by strong stimuli (e.g., PMA, pathogens, and immune complexes) and proceeds through (1) activation with robust ROS production, (2) chromatin decondensation driven in part by PAD4 activation and calcium signaling, (3) nuclear envelope breakdown and mixing of decondensed chromatin with cytoplasmic and granule proteins (including NE and MPO), and (4) plasma membrane rupture resulting in NET release and neutrophil death. Vital NETosis (**right**) can be induced by pathogens, platelets, or immune complexes and is characterized by (1) activation that is often NOX-independent, (2) vesicle formation and DNA export, (3) extracellular NET release without overt membrane rupture, and (4) preservation of neutrophil viability, allowing continued effector functions. Arrows indicate the direction of flow or progression. Created in using BioRender. Elisa Giovannetti (2026) (https://app.biorender.com/, accessed on 2 February 2026).

NETosis can be initiated by infection-related stimuli and can also be sustained in sterile inflammation, immunothrombosis, and tumor-associated chronic inflammation [43,44]. Platelet activation and platelet–neutrophil interactions represent important triggers, with pathways implicating toll-like receptor 4 (TLR4) and adhesion molecules such as P-selectin (CD62P) [43,46]. In the tumor-associated inflammation, multiple tumor-derived or tumor-induced factors can prime neutrophils toward a “NETosis-prone” state. These include granulocyte colony-stimulating factor (G-CSF), tumor-derived extracellular vesicles, and cytokine networks such as IL-8, IL-17, and TGF-β, which collectively enhance recruitment, activation, and oxidative stress to promote sustained NET release [18,43,44] (Figure 1). NETosis is a dynamic cascade orchestrated by multiple signaling modules. In suicidal NETosis, activation of the NOX2/NADPH oxidase axis and subsequent reactive oxygen species (ROS) generation is frequently considered an early upstream event that facilitates chromatin remodeling [18,43]. Peptidylarginine deiminase 4 (PAD4)-mediated histone citrullination is closely linked to chromatin decondensation in cancers; however, its requirement is stimulus- and phenotype-dependent and is not universal across all NET programs [45,47]. By comparison, vital NETosis is often ROS-independent or minimally ROS-dependent and can utilize vesicular trafficking to enable rapid DNA extrusion [48,49]. In chronic inflammatory, NETosis may further couple to amplification circuits: inflammatory transcriptional programs such as NF-κB upregulate cytokines and chemokines, and reciprocal reinforcement between the NLRP3 inflammasome and NETosis has also been proposed, forming positive feedback loops that favor persistent NET accumulation [48,49] (Figure 1).

Collectively, NETosis reflects neutrophil functional reprogramming under chronic inflammatory cues. Tumor-related signaling, platelet-mediated interactions, and cytokine networks can sustain NETosis and promote NET accumulation within the tumor milieu.

## 4. The Neutrophil and NET Axis as a Biomarker of Prognosis and Resistance to Immunotherapy in NSCLC

A growing body of evidence indicates that neutrophils and NETs are closely associated with the clinical outcomes of ICIs in NSCLC. Across multiple cohorts, the “neutrophil–NET axis” shows consistent correlations with treatment response, progression-free survival (PFS), and overall survival (OS) after ICI therapy [50,51,52]. These associations can be observed at several levels, including peripheral blood metrics such as NLR, absolute neutrophil count (ANC), and other myeloid related parameters; surrogate markers of circulating NETs such as citrullinated histone H3 (CitH3) and MPO–DNA complexes; and the density and spatial distribution of NETs within tumor tissues. Collectively, these readouts suggest biomarker potential for risk stratification and for identifying immune ecosystems that are less sensitive to immunotherapy or more prone to developing resistance [53,54,55].

### 4.1. Neutrophil-Related Metrics and ICI Outcomes

Peripheral inflammatory markers are inexpensive, readily accessible, and suitable for longitudinal monitoring, which makes them practical tools for assessing ICI efficacy in NSCLC. They are also commonly used as surrogate readouts of systemic immune status.

Among these, baseline NLR has the strongest and most consistent evidence, with multiple retrospective studies showing that NSCLC patients with elevated baseline NLR receiving ICIs often have shorter PFS and OS [50,51,52]. For example, a meta-analysis in advanced NSCLC treated with ICIs reported that high baseline NLR was significantly associated with increased risks of disease progression and death [50]. Similar findings have been reproduced across populations, commonly used thresholds, and lines of therapy, supporting NLR as one of the most widely applied and relatively robust peripheral prognostic indicators in this setting.

Beyond NLR, many studies have examined ANC, which reflects the overall circulating neutrophil burden, as well as derived NLR (dNLR), which is often used when absolute lymphocyte counts are unavailable and is commonly defined as dNLR = ANC/(WBC − ANC), where ANC denotes absolute neutrophil count and WBC denotes white blood cell count [54,56]. Meta-analytic evidence in NSCLC cohorts treated with ICIs further indicates that elevated baseline dNLR is also predictive of inferior survival outcomes [54]. In real-world cohorts, analyses that model NLR and ANC as continuous or stratified variables in Cox models similarly demonstrate significant associations between higher NLR and ANC with poorer survival [57]. Clinically, these findings converge on a common interpretation. A systemic inflammatory state dominated by myeloid activation, together with relative lymphocyte insufficiency, is often indicative of stronger immunosuppressive pressure and reduced likelihood of benefit from ICIs.

However, NLR and ANC are broad indicators of systemic inflammation and cannot distinguish functional states across myeloid subsets. In contrast, LDNs often exhibit more pronounced immunosuppressive features and may therefore more directly reflect a myeloid-suppressive immune context associated with primary resistance to PD-1 monotherapy [27,55]. Multiple studies from different angles suggest that when immunosuppressive neutrophil or PMN-MDSC subsets predominate in peripheral blood, early ICI response and survival outcomes are often worse. In a prospective first-line monotherapy with pembrolizumab, investigators used flow cytometry to define and dynamically track LDNs in peripheral blood. They found marked enrichment of baseline LDNs in patients with disease progression, whereas responders had few or no detectable LDNs. Receiver operating characteristic (ROC) analysis further proposed a baseline LDN percentage threshold of around 7%. When LDN% exceeded 7%, the objective response rate was 0%, and PFS and OS were significantly worse, accompanied by higher risks of rapid progression and early death [55]. This pattern, in which neutrophil or myeloid suppression dominance coincides with limited benefit from ICI monotherapy, aligns with observations from other peripheral myeloid-suppressive metrics. Kim et al. reported that peripheral immune cell proportions related to Tregs/Lox-1^+^ PMN-MDSCs predicted early responses to anti-PD-1 therapy [58]. Youn et al. further showed that the early post treatment NK/Lox-1^+^ PMN-MDSC ratio had good discriminative ability for response and was associated with PFS and OS [59]. In addition, Mezquita et al. reported in advanced NSCLC that persistently high dNLR and increased proportions of immature neutrophils by flow cytometry were associated with early ICI failure and poorer survival, suggesting that aberrant granulopoiesis and expansion of immature neutrophils may represent a key component of resistance [60,61].

Notably, the prospective LDN study also suggested that the association between high baseline LDNs and poor benefit from ICI monotherapy was substantially attenuated in patients receiving platinum-based chemotherapy plus pembrolizumab [55]. Under chemo-immunotherapy combinations, baseline LDNs were no longer clearly linked to efficacy, and some patients with high baseline LDNs still responded. Moreover, LDN levels decreased rapidly early during treatment, suggesting that combination regimens may partially reset, or at least weaken, the unfavorable myeloid-suppressive state represented by elevated baseline LDNs [55]. Taken together, LDNs may function more plausibly as a predictive biomarker for PD-1 monotherapy, reflecting a myeloid-suppressive immune environment that is difficult to reverse by checkpoint blockade alone, rather than as a purely prognostic marker of overall risk (Table 2).

### 4.2. NETs and ICI Outcomes

Compared with NLR and ANC, which primarily reflect systemic inflammatory burden, circulating NET markers such as CitH3, MPO–DNA, and cell-free DNA are more directly linked to neutrophil activation and NETosis. Accordingly, they have increasingly been evaluated for predicting ICI efficacy and prognosis in NSCLC [16,62]. Clinical evidence first emerged at the peripheral blood level. In a retrospective cohort of 185 patients with advanced or recurrent NSCLC treated with PD-1 or PD-L1 inhibitors, plasma CitH3, a surrogate of NETs, was measured before treatment and at 6 weeks after treatment initiation [16]. Elevated baseline CitH3 was associated with significantly shorter PFS and OS and remained an independent adverse factor after multivariable adjustment [16]. Outcomes were particularly poor among patients whose CitH3 remained high after treatment or increased further relative to baseline, suggesting that dynamic monitoring may provide additional information about an immune ecosystem characterized by persistent NET activity, associated with acquired resistance [16]. In addition, Guo et al. proposed that combining serum NET levels with CD8^+^ T-cell metrics and PD-L1 tumor proportion scores in a joint model could further improve the prediction of PD-1 inhibitor efficacy. This finding supports the notion that NETs are not merely byproducts of inflammation but may capture myeloid activation signals more proximal to immunotherapy response [62].

Intratumoral NET deposition and its spatial organization may more directly determine whether effector immune cells can access tumor nests, thereby shaping responses to immunotherapy [63,64]. In a first-line chemotherapy plus immunotherapy cohort, Lv et al. performed multiplex immunofluorescence on pretreatment biopsy specimens and quantified intratumoral NET density [63]. They found that high NET density was associated with poorer treatment response and significantly shortened PFS and OS. Spatial analyses further showed an overall negative association between NETs and CD8^+^ T-cell density and a positive trend with cancer-associated fibroblast (CAF)-related markers [63]. Outcomes were particularly poor when high-density CAFs and CD8^+^ clustering occurred within approximately 30 μm of NET-rich regions, which is consistent with an immune-excluded model. In this setting, T cells are present but are constrained to NETs and stroma-enriched boundary zones and have difficulty entering tumor cell nests to sustain effective cytotoxicity [63]. Consistently, Hong et al. examined tumor samples from 115 patients with advanced NSCLC receiving first-line immunotherapy and observed that high NET expression was associated with worse PFS and OS [64]. NETs also co-occurred with immunosuppressive TME features, showing negative correlations with CD8^+^ T-cell infiltration and positive correlations with Treg infiltration, further strengthening the tissue-level link between NETs, myeloid suppression, and limited benefit from immunotherapy [64] (Table 2).

### 4.3. NETs and Neutrophil Related Gene Signatures and Immunotherapy Response

Beyond cell counts and protein biomarkers, transcriptome-derived NETs and neutrophil-related gene signatures provide a scalable and reusable approach to characterizing immune ecosystems marked by NET activity and neutrophil enrichment. These signatures offer two practical advantages. First, they can be externally validated across public datasets such as TCGA and GEO, as well as in emerging clinical cohorts, which supports generalizability. Second, transcriptomic signals can integrate changes in myeloid inflammation, immunosuppression, and cell interaction pathways, enabling a more coherent framework that connects tumor subtypes, biological interpretation, and outcome prediction [65,66].

In NSCLC, multiple studies have constructed risk scores based on neutrophil/NET-related genes or lncRNAs and evaluated their relationships with immune infiltration patterns and predicted sensitivity to immunotherapy. For instance, Zhu et al. developed and validated a prognostic model based on neutrophil-related genes in lung adenocarcinoma, highlighting the feasibility of leveraging neutrophil-associated transcriptomic programs for risk stratification and immunologic characterization [67]. Similarly, studies using public datasets to develop NET-related gene signatures have reported worse survival in high-risk groups. Immune infiltration and pathway analyses often suggest enrichment of myeloid inflammation and immunosuppressive signaling in these groups, consistent with a “myeloid-dominated” TME that constrains T-cell effector function [65,66]. Such studies commonly integrate immune scores, checkpoint molecule expression, and deconvolution tools such as CIBERSORT and TMER to estimate immune cell composition [68,69]. Overall, NET-related signatures repeatedly align with the enrichment of neutrophils and myeloid-suppressive cells and are accompanied by reduced CD8^+^ T-cell infiltration or impaired function. At a systems level, these signatures tightly connect the neutrophil and NET axis with immunosuppressive or immune-excluded microenvironments. They provide quantifiable molecular evidence that helps to explain limited ICI benefit and establish a methodological foundation for cross-cohort validation and clinical translation [65,66] (Table 2).

**Table 2:** Neutrophil- and NET-related biomarkers for predicting ICI outcomes and resistance in NSCLC.

Biomarker	Sample Type	Assay/Measurement	Main Limitations	Potential Cut-Off(s)	References
NLR	Peripheral blood	Blood count	Low specificity (interference from infection/drugs)	4–6	[50,51,52,57]
ANC	Peripheral blood	Blood count	Low specificity (interference from infection/drugs)	9	[56]
dNLR	Peripheral blood	Blood count	Low specificity (interference from infection/drugs)	3	[54,60]
LDNs	Peripheral blood	Flow cytometry	Technically complex (flow cytometry), sample instability, lack of standardization	7	[55]
Tregs/Lox-1^+^ PMN-MDSCs ratio	Peripheral blood	Flow cytometry	Technically complex (flow cytometry), sample instability, lack of standardization	0.39	[58]
NK/Lox-1^+^ PMN-MDSC ratio	Peripheral blood	Flow cytometry	Technically complex (flow cytometry), sample instability, lack of standardization	5.75	[59]
Immature neutrophil proportion	Peripheral blood	Flow cytometry	Technically complex (flow cytometry), sample instability, lack of standardization	0.22%	[61]
CitH3	Plasma or serum	ELISA or immunoassay	High pre-analytical and assay variability (collection, processing, storage; kit/platform differences)	7	[16]
Intratumoral NET density	Tumor tissue	Multiplex immunofluorescence	Invasive (requires tissue), cannot monitor dynamically, affected by heterogeneity	1083 μm^2^	[63,64]
Neutrophil/NET gene signatures	Tumor tissue	Bioinformatic signature modeling	High cost, platform dependence, loss of spatial info, slow translation	NA	[65,66,67]

## 5. Mechanisms by Which the Neutrophil–NET Axis Drives Resistance to ICIs in NSCLC

Accumulating clinical evidence links neutrophils and NETs to the ICI response, PFS, and OS in NSCLC. However, the key critical question is mechanistic. Specifically, how does the neutrophil–NET axis disrupt the sequence of events required for effective checkpoint blockade, including effector T-cell priming, tumor infiltration, and sustained cytotoxicity, and ultimately manifest as primary or acquired resistance [16,63]. Current data suggest that this axis does not act at a single step. Instead, it shapes a resistance-prone ecosystem through coordinated effects at multiple levels. On one hand, neutrophils can shift toward suppressive states, including PD-L1^+^ TANs and PMN-MDSCs, and directly impair lymphocyte effector function through inhibitory mediators such as PD-L1, Arg-1, and ROS. On the other hand, NETs, together with their interactions with CAFs and the ECM, can establish structural and spatial immune exclusion that limits the access of CD8^+^ T cells and NK cells to tumor nests and reduces productive contact with tumor cells. In parallel, upstream inflammatory signaling pathways such as IL-6 and STAT3 and NF-κB and NLRP3, as well as specific molecular contexts such as STK11 and KEAP1-associated myeloid-dominant TME, can further amplify and stabilize these processes, thereby promoting the development and maintenance of ICI resistance in NSCLC [11,18,34,70].

### 5.1. Neutrophil-Driven Myeloid Immunosuppression Network

The failure of checkpoint blockade is not always explained by the absence of T cells in NSCLC. It is often associated with sustained dominance of myeloid immunosuppression. Clinically, the stable association between elevated peripheral NLR and ANC and poor ICI outcomes, together with observations that high LDN levels correlate with primary nonresponse to PD-1 monotherapy, suggests that enhanced systemic myeloid inflammation frequently corresponds to constrained effector immunity within tumors [55,71].

Within tumors, neutrophils appear as TANs and partially overlap with PMN-MDSCs in phenotype and function. In peripheral blood, LDNs are more often viewed as readouts of an immunosuppressive state [27,70]. These populations are not static in the chronic inflammatory milieu of NSCLC. They can be continuously reshaped by cytokines, hypoxia, and metabolic stress, progressively acquiring higher expression of suppressive molecules, heightened oxidative stress, and direct inhibitory capacity against lymphocyte effector functions [70]. In neutrophil-rich immune ecosystems linked to particular molecular contexts, myeloid suppression often coexists with reduced T-cell infiltration or impaired T-cell function, providing a permissive substrate for primary resistance to ICIs [33,34].

Immunosuppression is implemented through multiple parallel pathways. Inflammatory signaling in NSCLC can induce PD-L1 upregulation on neutrophils, enabling contact-dependent inhibition of T-cell and NK-cell activation and promoting reduced effector activity [72]. Sun et al. showed that TANs suppress NK-cell cytotoxicity through PD-L1/PD-1-dependent, cell–cell contact mechanisms in tumor-bearing mice, providing direct evidence that neutrophil PD-L1 can restrain innate effector function beyond tumor-cell PD-L1 alone [73]. Importantly, myeloid suppression does not rely on a single checkpoint axis. PMN-MDSCs and TANs can restrict T-cell proliferation and T-cell receptor signaling through Arg-1 mediated arginine depletion. Steggerda et al. demonstrated that pharmacologic arginase inhibition (CB-1158) reverses Arg1-driven suppression and synergizes with checkpoint blockade across mouse tumor models, consistent with arginine depletion acting as a metabolic “brake” that limits the functional recovery of T cells during ICI therapy [74]. In addition, PMN-MDSCs and TANs can drive oxidative stress through ROS and RNS, thereby promoting functional-exhaustion-like states. They can also release mediators such as PGE2 that further lower the threshold for effective effector immunity [70]. As a result, in NSCLC with highly active myeloid suppression, even when PD-1 and PD-L1 are blocked, T cells may still fail to regain effective cytotoxicity because of metabolic and oxidative constraints, yielding a resistance phenotype in which checkpoint inhibition occurs but effector function does not recover [70]. Myeloid suppression is further amplified and stabilized through intercellular interactions. Neutrophil-associated suppressive populations not only act directly on effector lymphocytes but also inhibit dendritic cell (DC) maturation and antigen presentation, thereby reducing the efficiency of initial T-cell priming. They can promote expansion of Tregs or enhance their suppressive function, which further constrains effector immunity. They also form mutually reinforcing immunosuppressive networks with TANs and fibroblasts [70]. At the same time, the persistence of these suppressive myeloid programs is supported by continuous replenishment, which may help to explain the clinical observation that a subset of patients with NSCLC experience rapid progression early during immunotherapy [63,75].

**Figure 2:**
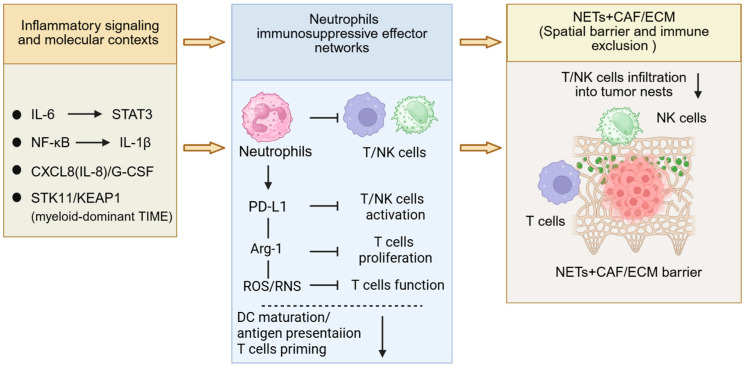
Neutrophil–NET axis drives resistance to ICIs in NSCLC. Inflammatory signaling and molecular contexts, including IL-6/STAT3 activation, NF-κB–IL-1β signaling, CXCL8 (IL-8)/G-CSF–driven myeloid recruitment, and STK11/KEAP1 alterations associated with a myeloid-dominant TME, promote the establishment of immunosuppressive neutrophil effector networks. These neutrophil programs suppress antitumor immunity via PD-L1 upregulation, arginase-1 (Arg-1) activity, and increased reactive oxygen/nitrogen species (ROS/RNS), thereby impairing T/NK-cell activation, proliferation, and effector function, and potentially limiting dendritic cell (DC) maturation, antigen presentation, and T-cell priming. In parallel, NET deposition together with CAFs and ECM remodeling forms a spatial barrier that restricts T- and NK-cell infiltration into tumor nests, reinforcing immune exclusion and contributing to reduced sensitivity to immune checkpoint blockade. Horizontal arrows indicate activation or signaling flow; blunt-ended lines indicate inhibitory effects; downward arrows indicate decreased levels, impaired function, or reduced infiltration. Created in BioRender. Elisa Giovannetti (2026) (https://app.biorender.com/, accessed on 2 February 2026).

Taken together, neutrophil-associated suppressive subsets constitute a key effector network for ICI resistance in NSCLC. They inhibit effector lymphocytes through multiple pathways, including PD-L1, Arg-1, and ROS, and they establish amplification loops at the levels of antigen presentation and immune regulation. Consequently, even when some degree of immune infiltration is present, it may not translate into effective antitumor immunity [63,70,72] (Figure 2).

### 5.2. Spatial Barriers and Immune Exclusion

Clinical benefit from ICIs depends to a large extent on whether effector immune cells can enter tumor nests and establish stable cytotoxic contacts with tumor cells in NSCLC [76,77]. A substantial fraction of NSCLC exhibits an immune-excluded spatial configuration. CD8^+^ T cells are not absent, but are preferentially retained within stroma or at the tumor margin and have difficulty traversing the matrix to access tumor nests. This pattern, in which immune cells reach the boundary but not the core, means that even if PD-1 and PD-L1 blockade removes inhibitory signaling, the spatial prerequisites for sustained killing may be lacking, resulting in primary or early resistance [76,77,78,79].

The spatial substrate of immune exclusion is often established by CAF-driven ECM deposition and remodeling. Stromal densification and mechanical barriers reshape migration routes and are accompanied by immunoregulatory signals that favor effector T-cell retention in stromal regions [79,80,81]. Spatial studies in lung cancer further suggest that specific CAF programs are closely linked to T-cell marginalization, providing a histologic explanation for tumors with tumor-infiltrating lymphocytes yet insufficient cytotoxic activity [78,81]. Within this stromal framework, NETs may further reinforce spatial constraints. NETs comprise extracellular DNA and protein webs with adhesive and mesh-like trapping properties. They can deposit around tumor nests or within stromal tracks, increase migratory resistance for effector cells, and reduce the efficiency with which CD8^+^ T cells and NK cells form immune synapses and execute localized cytotoxicity [82,83,84]. Mechanistic studies across tumor types support the concept that NETs can coat tumor cells and weaken contact-dependent killing by cytotoxic lymphocytes, whereas promoting NET degradation or inhibiting NET formation can enhance immune effector function and synergize with checkpoint blockade [82,83,84]. Chen et al. reported in a lung cancer model that reducing NET formation/accumulation was accompanied by enhanced anti-PD-1 antitumor activity and improved intratumoral CD8^+^ T-cell effector function, supporting NETs as a modifiable barrier to checkpoint responses in vivo [85]. In addition, Deng et al. showed that neutrophil-specific PAD4 deletion or pharmacologic PAD4 inhibition diminished NETosis and substantially enhanced ICI Immune checkpoint inhibitors efficacy across mouse tumor models, reinforcing PAD4–NETosis as a mechanistic entry point to overcome ICI resistance [86].

Overall, the CAF and ECM axis provides a stromal framework that is difficult to penetrate, and NETs add local structural obstacles that limit contact and effective killing. Together, they constitute a key spatial basis for ICI resistance in NSCLC (Figure 2).

### 5.3. Inflammatory Signaling and Molecular Contexts That Stabilize ICI Resistance in NSCLC

The immunosuppression related to the neutrophil–NET axis is rarely a one-time event in NSCLC. Conversely, it often manifests as a durable and self-sustaining microenvironmental state [76,84,87]. A central premise for this stabilization is persistent upstream inflammatory signaling that forms positive feedback loops, together with tumor-specific molecular contexts that provide selective advantages for maintaining a myeloid-dominant TME. The net result is a stable shift toward a low-responsiveness state characterized by insufficient effector priming, restricted infiltration, and progressive functional attenuation. Under these conditions, PD-1 and PD-L1 blockade is less likely to be translated into sustained effective antitumor immunity [76,87,88].

IL-6 and STAT3 signaling is widely considered a key hub for sustaining myeloid immunosuppression. In NSCLC, IL-6 derived from tumor cells and stromal cells can chronically activate STAT3 in myeloid cells, promoting expansion and maintenance of suppressive myeloid populations. This is accompanied by upregulation of immunosuppressive molecules and increased activation thresholds for effector lymphocytes [87,88]. In this context, suppressive phenotypes such as PD-L1^+^ TANs and PMN-MDSCs are more likely to persist, thereby limiting restoration of T-cell function after checkpoint blockade. For instance, Jeong et al. showed that tumor-intrinsic signaling can amplify an IL-6–JAK–STAT3 program that drives MDSC accumulation and dampens CD8^+^ T-cell effector function; in a murine lung cancer model, combined IL-6 blockade with anti-PD-1 improved tumor control, concomitant with reduced MDSCs and increased granzyme B^+^/IFNγ^+^ CD8^+^ T cells in vivo [88]. In addition, NF-κB and NLRP3 inflammasome-related pathways link tissue damage signals to amplification of chronic inflammation. PRR-mediated NF-κB activation drives transcription of multiple inflammatory mediators and provides priming signals that facilitate inflammasome assembly. Inflammasome activation then generates factors such as IL-1β, which further strengthens local inflammation and myeloid recruitment and can couple with tissue remodeling, epithelial–mesenchymal transition, and immune escape programs [89,90,91]. Chen et al. reported that disrupting a PIM1/NF-κB/CCL2 inflammatory program reshaped macrophage infiltration/polarization and enhanced responses to anti-PD-1 therapy in NSCLC, reinforcing the notion that NF-κB-coupled inflammatory chemokine output can constrain effective antitumor immunity during ICI treatment [92]. With respect to NET-associated processes, these pathways can serve as upstream sources that induce and maintain NETosis, and they can form amplification loops when damage-associated components released during NET formation further potentiate inflammatory signaling. This coupling can increase the stability of an immunosuppressive state [84,89,90,91].

Beyond these pathway hubs, chemotactic and hematopoietic mobilization signals determine whether a myeloid-dominant microenvironment can be continuously replenished. In particular, common chemokine axes such as CXCL8-related signaling and granulocyte-mobilizing factors such as G-CSF can promote neutrophil recruitment and emergency granulopoiesis, maintaining a high flux of suppressive myeloid cells into lung tumors [75,93,94]. This supply-side mechanism implies that even if therapy temporarily reduces local suppressive components, persistent systemic mobilization can rapidly repopulate the tumor, thereby prolonging immunosuppressive ecosystems over time and providing microenvironmental conditions for acquired resistance [94,95]. Horn et al. further demonstrated that inhibiting CXCR1/2-dependent myeloid trafficking reshaped the tumor microenvironment (including reduced granulocytic MDSC infiltration), increased T-cell infiltration, and improved checkpoint-based antitumor activity in murine models of lung cancer [96]. Certain genomic subtypes of NSCLC are more likely to establish neutrophil-rich, myeloid-dominant TME. In KRAS-mutated contexts, STK11 inactivation frequently co-occurs with reduced immunogenicity, limited T-cell infiltration, and enhanced myeloid inflammation, suggesting that microenvironmental remodeling can reduce dependence on PD-1 and PD-L1 signaling for immune escape [32,97]. Skoulidis et al. demonstrated that Stk11/Lkb1 deficiency confers resistance to PD-1/PD-L1 blockade, supporting a mechanistic link between this genotype, myeloid-dominant inflammation, and primary ICI resistance in KRAS-mutant murine lung cancer models [32]. KEAP1 alterations influence tumor cell states and tumor–microenvironment interactions through programs related to oxidative stress responses and metabolic adaptation, which may facilitate persistence of immunosuppressive phenotypes. In these contexts, closed loops comprising recruitment, maintenance of suppressive phenotypes, inflammatory amplification, and tissue remodeling are more readily established and stabilized, manifesting clinically as low primary sensitivity to ICIs or early acquired resistance [32,34,35,98].

Overall, maintenance of ICI resistance in NSCLC is consistent with the emergence of a self-consistent steady state. Persistent inflammatory signaling provides driving forces and amplification. Continuous myeloid replenishment sustains the state over time. Tumor molecular contexts stabilize this configuration under selective pressure. The neutrophil–NET axis occupies a central position within this network. It responds to upstream inflammatory inputs and reinforces suppressive ecosystems through feedback mechanisms, offering an integrated microenvironmental explanation for limited efficacy and persistent resistance to checkpoint blockade (Figure 2).

## 6. Targeting the Neutrophil and NET Axis to Overcome ICI Resistance in NSCLC

As the importance of neutrophils and NETs in the NSCLC immune microenvironment has become increasingly recognized, pharmacologic interventions targeting this axis have emerged as attractive translational directions across multiple disease models [82,99,100]. Available evidence indicates that blocking chemotactic signals that govern neutrophil recruitment, or modulating suppressive neutrophil states, can alleviate neutrophil-mediated immunosuppression and enhance responses to immunotherapy in diverse tumor models. In parallel, direct interference with NET formation or stability has been shown to mitigate tissue injury and protumor effects and, in certain models, improve sensitivity to immune checkpoint blockade [82,99,101].

### 6.1. Inhibiting Neutrophil Recruitment or Polarization in NSCLC

In myeloid-dominant TME in NSCLC, sustained neutrophil accumulation and maintenance of suppressive phenotypes typically depend on persistent inputs from chemokine networks and upstream inflammatory or stress signals. From an intervention perspective, two actionable steps are most commonly targeted. The first approach is to reduce the inflow of neutrophils into tumors through recruitment and trafficking. The second approach is to weaken phenotypic skewing toward suppressive TANs or PMN-MDSC-like states within the tumor milieu through polarization and functional reprogramming. Both strategies aim to shift neutrophil-rich immune ecosystems toward conditions that better support effector T-cell priming, infiltration, and sustained cytotoxicity, thereby improving the inducibility and durability of ICI effects [82,102,103].

The CXCL8-related CXC chemokine axis is one of the principal pathways targeted to suppress neutrophil recruitment and migration and has become a common translational entry point for “de-myeloid” strategies combined with ICIs [82,102]. In NSCLC, CXCL8 and related CXC chemokines promote tumor entry of neutrophils and PMN-MDSCs and are associated with the maintenance of myeloid-suppressive phenotypes. Accordingly, antagonists targeting CXCR1 and CXCR2, or CXCR2 alone, have been incorporated into combination designs with ICIs [82,102]. Representative agents include the CXCR1/2 inhibitor SX-682, which has shown early clinical activity in metastatic melanoma when combined with pembrolizumab, while its utility in NSCLC is currently under investigation in a phase II trial in combination with pembrolizumab for metastatic or recurrent stage IIIC/IV disease, with feasibility, pharmacodynamic myeloid modulation, and preliminary efficacy as key objectives [97,104]. In contrast, the CXCR2 antagonist navarixin (MK-7123/SCH-527123) has been evaluated with pembrolizumab in a randomized phase II multitumor program (including NSCLC), but the combination was closed at a prespecified interim analysis for insufficient efficacy despite manageable safety [105].

The complement-associated C5a–C5aR1 inflammatory chemotaxis axis represents another actionable pathway to limit neutrophil activation, migration, and myeloid repopulation and provides a translational option with available pharmacologic tools to attenuate inflammation-driven myeloid input [106,107,108]. As a potent neutrophil chemoattractant and activator, C5a can promote neutrophil replenishment and help to sustain immunosuppression in tumor-associated inflammation. Clinically, this pathway has been interrogated at the receptor level in combination with checkpoint blockade. In the phase I STELLAR-001 study enrolling advanced solid tumors (including NSCLC), the anti-C5aR1 antibody avdoralimab (IPH5401) plus durvalumab was well tolerated, but minimal antitumor activity in the expansion cohorts led to early termination of the study [109]. The anti-C5a antibody vilobelimab (IFX-1) has demonstrated acceptable safety and feasibility in a published phase II setting outside oncology (severe COVID-19), supporting the druggability of C5a blockade; however, oncology-focused efficacy data, including ICI-combination cohorts, remain limited and largely early-stage, and NSCLC-specific efficacy signals are not yet established [107].

Key signaling hubs such as TGF-β and IL-6 and STAT3 are major upstream drivers that induce and stabilize TANs and PMN-MDSC-like suppressive phenotypes. They therefore constitute central translational entry points for blocking polarization or functional skewing and for remodeling myeloid outputs [103,110,111]. Unlike strategies that aim to deplete neutrophils, these approaches emphasize altering functional programs so that neutrophil cells no longer persistently execute immunosuppressive activities. Bintrafusp alfa (M7824), a bifunctional fusion protein designed to block PD-L1 while trapping TGF-β, demonstrated early signals with a manageable safety profile in previously treated NSCLC, but a later randomized phase III comparison in PD-L1-high advanced NSCLC did not show superiority over pembrolizumab, underscoring the need for biomarker-enriched selection and optimized combinations [112,113]. For STAT3-directed modulation, the HUDSON phase 2 umbrella trial in immunotherapy-resistant NSCLC included a durvalumab plus danvatirsen, a STAT3-targeting antisense oligonucleotide; overall, the reported response rates in this module were low [114]. For IL-6-directed modulation, a phase II trial is evaluating tocilizumab (anti IL-6R) combined with nivolumab plus ipilimumab to attenuate IL-6 STAT3-driven myeloid inflammation, focusing on safety and translational immune readouts in NSCLC [115].

### 6.2. Direct Targeting of NETs

Compared with upstream approaches that target neutrophil recruitment, direct targeting of NETs focuses more on their physical structures and downstream effects within the TME. As DNA and protein webs, NETs can spatially restrict immune cell migration and tumor cell contact. They can also carry or concentrate inflammatory and immunoregulatory molecules, thereby shaping locally entrenched immunosuppressive ecosystems that are difficult to reverse [82,99]. Accordingly, direct anti-NET strategies usually pursue two objectives, reducing existing NET burden or inhibiting NETosis to decrease ongoing production [99,100,101,116].

Deoxyribonuclease (DNase)-mediated degradation of the DNA backbone is the most straightforward approach to reducing NET burden and has become one of the most widely used translational entry points for NET targeting [99]. Clinically, the most established DNase agent is recombinant human DNase I (dornase alfa), which has been repurposed in inflammatory lung disease settings with clear pharmacodynamic evidence of NET degradation [99]. For example, in severe COVID-19 pneumonia, nebulized dornase alfa reduced inflammatory markers and NET-associated readouts (including decreases in DNA–MPO complexes) and was associated with improved clinical recovery metrics in a randomized trial framework, supporting feasibility and target engagement in humans [117]. In cancer, DNase-based NET disruption is still being evaluated primarily in proof-of-concept settings [118]. For example, an ongoing phase II study is testing dornase alfa in combination with cisplatin in refractory germ cell tumors to assess whether dismantling extracellular DNA/NET scaffolds can enhance therapeutic responsiveness [118].

The PAD4-mediated histone citrullination and chromatin decondensation axis is a key molecular step in NETosis and provides a core pharmacologic entry point for source control of NET formation [116]. Clinically, however, PAD4-directed translation is still emerging and oncology-focused efficacy readouts are not yet available. The most mature human data to date come from early first-in-human programs targeting PAD enzymatic activity. For example, AZD1163, a bispecific antibody designed to inhibit extracellular PAD2/4 activity, has reported phase I results in healthy volunteers showing good tolerability, favorable pharmacokinetics, and clear target engagement as measured by dose-dependent inhibition of PAD2/4 activity ex vivo [119]. In parallel, a phase II study of AZD1163 in rheumatoid arthritis is ongoing, reflecting continued clinical development of PAD pathway inhibition [120].

In addition to PAD4 enzymatic blockade, neutrophil elastase (NE) participates in chromatin remodeling and granule protein translocation in selected NETosis programs. In a randomized perioperative study, sivelestat significantly reduced postoperative inflammatory and immunosuppression-associated markers, including lower IL-6 early after surgery and lower IAP at later postoperative time points, compared with controls [121]. AZD9668 (alvelestat), an oral neutrophil elastase inhibitor, was evaluated in a published phase II randomized study in bronchiectasis. Four weeks of treatment was associated with an improvement in lung function, with an approximate 100 mL increase in FEV1, and showed trends toward reductions in sputum inflammatory biomarkers, with overall good tolerability [122].

Overall, a broad range of neutrophil-directed strategies has been developed, and preclinical studies in NSCLC have already tested several of these approaches in combination with ICIs, with some showing encouraging synergy and improved antitumor responses. In contrast, although direct NET-targeting modalities (e.g., NET degradation or inhibition of NETosis) are conceptually compelling and supported by translational evidence in other disease contexts, they have not yet been specifically established in NSCLC, nor is there clear, disease-focused evidence for their combination with ICI therapy.

## 7. Conclusions and Future Perspectives

In summary, current clinical and mechanistic evidence converges on a relatively coherent resistance mechanism in NSCLC, with neutrophil enrichment and phenotypic skewing, while NET formation and deposition are coupled with sustained activation of inflammatory signaling programs, including NF-κB and NLRP3 and IL-6 and STAT3. Together, these processes drive the TME toward immunosuppression and immune exclusion, thereby diminishing ICI benefit and facilitating primary or acquired resistance. Along this axis, multiple layers of readouts have translational potential. Peripheral LDNs may help to identify high-risk populations for PD-1 monotherapy. Circulating NET biomarkers, including CitH3 and MPO–DNA complexes, may support risk stratification and dynamic monitoring. Tissue NET density and spatial organization, together with TAN- and NET-related gene signatures, may enable definition of neutrophil and NET-rich immune subtypes and more refined patient stratification. Correspondingly, interventions that block recruitment through the IL-8 and CXCR1 and CXCR2 axis, strategies that inhibit or dismantle NETs via PAD4 inhibition or DNase treatment, and approaches that reprogram myeloid states provide clear mechanistic routes for ICI-based combinations to overcome resistance. These efforts must also account for the physiological roles of neutrophils and NETs in host defense, which highlights the importance of biomarker-guided enrichment, stratified implementation, and careful management of safety windows.

A central priority is to translate associations into causal, clinically actionable tools. First, biomarker validation should be stepwise: retrospective discovery and cut-off selection, independent external validation, and prospective confirmation in ICI-treated NSCLC. Standardization of pre-analytical handling, assay platforms, and reporting is essential for circulating NET surrogates (e.g., CitH3, MPO–DNA) and neutrophil-state measures (e.g., LDNs by flow cytometry), with pre-specified thresholds and head-to-head benchmarking against PD-L1, tumor mutational burden, and circulating tumor DNA. Studies should also separate prognostic vs. predictive value using appropriate comparators (e.g., ICI monotherapy vs. chemo-immunotherapy) and incorporate dynamic assessments (baseline and early on-treatment, ~2–6 weeks) to capture evolving resistance. Second, multi-omics integration should enable standardized, spatially resolved subtyping. Combining single-cell profiling with spatial transcriptomics, multiplex imaging, and digital pathology can quantitatively define TAN programs and NET-rich architectures, including spatial relationships with CAFs and CD8^+^ T cells. A practical goal is to develop reproducible NET-high vs. NET-low immune subtypes using locked feature sets (cell densities, spatial proximity metrics, and transcriptomic signatures) and link them to molecular contexts (e.g., STK11/KEAP1), immune exclusion, and outcomes. Portability requires multi-center training/validation, batch-effect control, and transparent performance reporting. Third, clinical trials should embed mechanism-based stratification and pharmacodynamics from the outset. Combination trials targeting neutrophil recruitment/polarization or NET burden should predefine enrichment (myeloid-dominant TME, NET-high markers, elevated LDNs, and immune-excluded patterns), incorporate serial biospecimen collection, and include pharmacodynamic endpoints demonstrating on-target remodeling (NET burden, neutrophil states, CD8^+^ access to tumor nests) alongside objective response rate/PFS and durability. Biomarker-enriched phase Ib/II designs can optimize dosing, sequencing, and safety.

Ultimately, coordinated biomarker validation, spatial multi-omics classification, and biomarker-embedded trials are required to move the neutrophil–NET axis toward practical stratification and therapeutic strategies for ICI resistance in NSCLC.

## Figures and Tables

**Table 1:** Phenotypic and functional characterization of neutrophil subsets in NSCLC.

Neutrophil Subset	Key Markers	Localization	Characteristics
HDNs	CD11b^+^, CD15^+^/CD66b^+^, CD14^−^, CD16, CD10^+^	Predominantly in blood, with minimal infiltration in tumor tissue	Mature neutrophils with potent antimicrobial and phagocytic activity under homeostasis; exert intrinsic antitumor activity in the tumor setting
LDNs	CD11b^+^, CD15^+^/CD66b^+^; CD14^−^, PBMC fraction; CD10^−^/CD16, PDL1	Predominantly in blood, with minimal infiltration in tumor tissue	Highly heterogeneous population; predominantly displays protumor and immunosuppressive phenotypes in cancer, and can inhibit T-cell proliferation and activation
PMN-MDSCs	CD11b^+^, CD14^−^, CD15^+^, CD66b^+^, HLA-DR^−^, LOX-1^+^, ARG1^+^, iNOS^+^, CD33^+^	Abundantly present in both blood and tumor tissue	Core feature is potent T-cell immunosuppression; promotes tumor angiogenesis, invasion, metastasis and immune escape
TAN-N1	No universal markers; reported ICAM-1 and MHC-II (context-dependent)	Exclusively present in tumor tissue	Anti-tumor phenotype in the tumor microenvironment; directly kills tumor cells, promotes CD8^+^ T-cell activation, with negligible immunosuppressive activity
TAN-N2	No universal markers; often ARG1 and PD-L1 inducible (context-dependent)	Exclusively present in tumor tissue	Protumor phenotype in the tumor microenvironment; potently suppresses T- and NK-cell function, drives tumor angiogenesis, invasion and distant metastasis

## Data Availability

Not applicable.

## Abbreviations

The following abbreviations are used in this manuscript:

ANC	Absolute neutrophil count
CAFs	Cancer-associated fibroblasts
DC	Dendritic cell
DNase	Deoxyribonuclease
dNLR	Derived neutrophil-to-lymphocyte ratio
ECM	Extracellular matrix
G-CSF	Granulocyte colony-stimulating factor
HDNs	High-density neutrophils
ICIs	Immune checkpoint inhibitors
LDNs	Low-density neutrophils
MDSCs	Myeloid-derived suppressor cells
MPO	Myeloperoxidase
NETs	Neutrophil extracellular traps
NE	Neutrophil elastase
NLR	Neutrophil-to-lymphocyte ratio
NOX	NADPH oxidase
NSCLC	Non-small cell lung cancer
OS	Overall survival
PD-1	Programmed cell death protein 1
PD-L1	Programmed death-ligand 1
PFS	Progression-free survival
PMN-MDSCs	Polymorphonuclear myeloid-derived suppressor cells
ROS	Reactive oxygen species
TANs	Tumor-associated neutrophils
TAN-N1	Tumor-associated neutrophils N1 phenotype
TAN-N2	Tumor-associated neutrophils N2 phenotype
TME	Tumor microenvironment
Tregs	Regulatory T cells
WBC	White blood cell
